# Temporal phytoremediation potential for heavy metals and bacterial abundance in drainage water

**DOI:** 10.1038/s41598-022-11951-w

**Published:** 2022-05-17

**Authors:** Mohamed Azab El-Liethy, Mohammed A. Dakhil, Ali El-Keblawy, Mohamed Abdelaal, Marwa Waseem A. Halmy, Abdelbaky Hossam Elgarhy, Ilunga Kamika, Ghada A. El-Sherbeny, Mai Ali Mwaheb

**Affiliations:** 1 Environmental Microbiology Laboratory, Water Pollution Research Department, National Research Centre, Dokki, Giza, 12622 Egypt; 2 Botany and Microbiology Department, Faculty of Science, Helwan University, Cairo, 11795 Egypt; 3 Department of Applied Biology, Faculty of Science, University of Sharjah, P.O. Box 27272, Sharjah, United Arab Emirates; 4 Botany Department, Faculty of Science, Mansoura University, Mansoura, 35516 Egypt; 5 Department of Environmental Sciences, Faculty of Science, Alexandria University, P.O. Box 21511 Alexandria, Egypt; 6 Central Laboratory for Environmental Quality Monitoring (CLEQM), National Water Research Center (NWRC), P.O. Box 13621/6, El-Kanater, Egypt; 7 Institute for Nanotechnology and Water Sustainability, College of Science, Engineering and Technology, University of South Africa, Florida, Johannesburg, 1709 South Africa; 8 Botany Department, Faculty of Science, Fayoum University, Faiyum, 63511 Egypt

**Keywords:** Environmental sciences, Pollution remediation, Plant ecology

## Abstract

Drainage water in developing countries has a high abundance of pathogenic bacteria and high levels of toxic and mutagenic pollutants. Remediation of drainage water is important in water-poor counties, especially with the growing need to secure sustainability of safe water resources to fulfill increasing demands for agriculture. Here, we assess the efficiency of macrophyte *Pistia* *stratiotes* to remediate a polluted drain in Egypt, rich in macronutrients, heavy metals, and different types of pathogenic and non-pathogenic bacteria. Drainage water was sampled monthly, for a year, to assess seasonal changes in bacterial abundance, water physicochemical properties (transparency, temperature, dissolved oxygen, EC, pH, N, P, and K), and heavy metals contents (Pb, Zn, and Co) in a polluted drain dominated with *P.* *stratiotes*. The ability of *P.* *stratiotes* to rhizofiltrate the three heavy metals was calculated. The results showed seasonal variations in the plant rhizofiltration potential of Co and *Salmonella* abundance. The highest values of dissolved oxygen (12.36 mg/L) and macronutrient elements (N and P) were attained in the winter. The counts of total coliform, fecal coliform, fecal streptococci, and in *Salmonella* spp. were the highest in the summer. *P.* *stratiotes* accumulated Pb more than Zn and Co. The highest levels of rhizofiltration were in summer for Pb and Co and in the autumn for Zn. Canonical correspondence analysis (CCA) showed that the variation in the bacterial abundance and plant rhizofiltration potential was strongly and significantly affected by water-dissolved oxygen. Moreover, the rhizofiltration potential of Pb and Co showed a positive correlation with water N. Overall, *P.* *stratiotes* could be proposed as a potential biomonitor for heavy metals in polluted water.

## Introduction

By 2050, water demands are expected to increase by four-hundred times for manufacturing and by one-hundred thirty times for household usage. In addition, about half of the world’s population will suffer from water shortage, even in river basin countries^1^. The available water resources in Egypt are from the River Nile (55.5 billion cubic meters (BCM)/Year), shallow groundwater (6.5 BCM/Year) and deep groundwater (2.4 BCM/Year) from the Western Desert and Sinai, and rainfall in the northern parts of the country (1.6 BCM/Year). Therefore, the total water supply is approximately 66 BCM/Year, while the current water demand is about 79.5 BCM/Year. Agriculture alone consumes about 85% of the country’s water^2^. The gap between water availability and demand has reached 13.5 BCM/Year, and it is continuously increasing due to population growth, urbanization, and industrial expansion. Due to these challenges, there is a need for reusing partially treated or untreated drainage water for agricultural purposes, either formally or informally^3,4^. Currently, the amount of drainage water that is reused is more than 9.0 BCM/Year, and it is expected to increase by 2050^5^. The River Nile receives wastewater discharged from about 74 agricultural and industrial drains^6^. For instance, Gaber et al.^7^

Drainage water contains huge amounts of pollutants such as solids, and organic and inorganic materials in soluble and insoluble forms, and heavy metals. These pollutants may be toxic and/or mutagenic^8,9^. Heavy metals such as zinc (Zn), cobalt (Co), copper (Cu), cadmium (Cd), chromium (Cr), iron (Fe), nickel (Ni), and lead (Pb) are usually found in industrial and domestic wastewater and drainage water^10^. Consumption of heavy metals polluted water can cause severe human health problems such as metabolic malfunction and damage to organs such as the liver; damage to the nervous system and bones; and, in severe cases, can cause cancer^4,11^. Many heavy metals, such as Fe, Cr, Cu, and Zn, are essential micronutrients at low concentrations for human. However, some of them can become toxic at high concentrations^12^. The disposal of partially treated and untreated wastewaters into drains for agricultural reuse negatively impacts human health and the environment because they contain microbial pathogens^13,14^. Thermotolerant coliform and pathogenic bacteria, viruses, and parasites such as *Salmonella*, *Vibrio*, *Pseudomonas*, *Mycobacterium*, *Listeria*, *Campylobacter*, adenovirus, and rotavirus are common in insufficiently treated and untreated wastewaters and their receiving drains^15–17^.

Continuous changes in natural and anthropogenic activities cause seasonal changes in surface water quality^18^. For example, temperature has a potential effect on water ecosystem health and can also accelerate organisms’ biochemical reactions and metabolic activities. In addition, the interactions of pollutants and microbial pathogens with other water inhabitants are affected by temperatures. Temperature variations commonly depend on climatic conditions, sampling times, and the number of sunshine hours^16,19^.

Conventional wastewater and drainage water purification technologies that remove heavy metals and microbial pollutants are frequently time consuming, environmentally destructive, and mostly inefficient^20,21^. Various technologies are used for heavy metals removal. These include reverse osmosis (RO)^22^, ion exchange^23^, chemical precipitation (^24^, photocatalytic oxidation^25^, and the application of nano-materials disinfectants^26,27^. However, there are some drawbacks associated with such technologies, including operational and maintenance costs, and some of them are not eco-friendly^28,29^. Phytoremediation, however, has been proposed as an effective, low cost and ecofriendly green technology for toxic metal removal from polluted water^4,30^.

Rhizofiltration, as a kind of phytoremediation technique, involves using plants in absorption, concentration, and/or precipitation of the heavy metals. Roots play an important role in the rhizofiltration process^31,32^. Rhizofiltration has been considered an appropriate approach for the removal of heavy metals and pathogenic microbes. For example, Odinga et al.^33^ assessed the ability of a constructed rhizofiltration system in the remediation of municipal wastewater and found a significant reduction in heavy metals and intestinal pathogens. The rhizofiltration system successfully removed between 45 and 98% of the detected pathogens. Environmental factors, such as pH in the rhizosphere and root exudates, can accelerate the precipitation of heavy metal on the surface of roots^34^. *P.stratiotes* (water lettuce) is a common invasive aquatic macrophyte in the water bodies of Egypt. This fast-growing plant has a great potential to remove macronutrients^35^ and heavy metals^30,36^. The objective of the current study was to assess the efficiency of *P.* *stratiotes* to reduce polluted drain in Egypt rich in macronutrients, heavy metals, and different types of pathogenic and non-pathogenic bacteria. In addition, the study evaluates relationships between seasonal variations in environmental factors on the one hand and chemical and microbial pollutants in drains on the other hand. The removal of the heavy metals and macronutrients that cause eutrophication and toxic heavy metals could reduce environmental, animal, and human health risks.

## Materials and methods

### Study area

**Figure 1:**
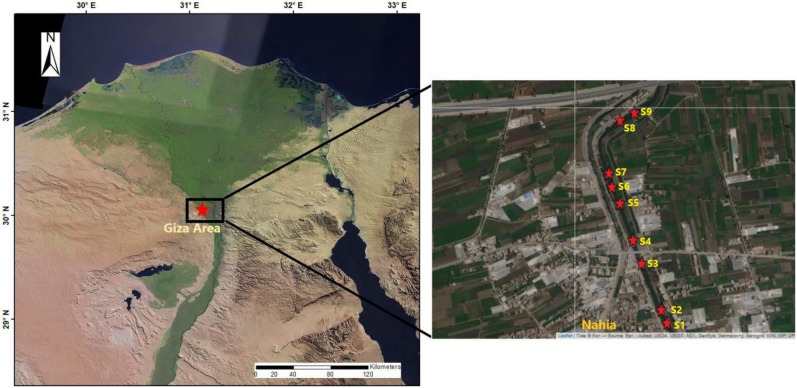
Study area showing Al-Labene Drain (red star) in Nahia, Giza Area. Sampling site (**S1**–**S9**) locations are indicated with small red stars.

The study drain is one of the drainage water plants in Nahia Area (Giza Governorate), namely Al-Labene Drain (Fig. 1). This drain is 11 km in length and receives untreated and partially treated municipal wastewater that is produced by the Abou-Rawash wastewater treatment plant (WWTP) through Al Barakat and Al-Ramal Drains, and it discharges effluent from the Al-Labene Drain into the El-Rahawy Drain. The biological oxygen demand (BOD) load of the Al-Labene drain is 15 Ton/day. Moreover, this drain receives nutrients and heavy metal pollutants from agricultural and industrial sources. Abou-Rawash WWTP receives 1,450,000 m^3^/day of raw municipal wastewater. However, there is about 200,000 m^3^/day that exceeds the plant’s capacity and is directly discharged to the Barakat Drain, then to Al-Ramal, Al-Labene, Al-Mariotya, and El-Rahawy Drains until they reach the Rosetta Branch without any treatment. Therefore, these drains receive the entire wastewater amount. The study site was located at the Al-Labene Drain at Giza area (Lat. 30.06147, Long. 31.12599). Landsat-7 satellite image for the study area was obtained and used to generate a map for the study area (Fig. 1) using ESRI ArcGIS 10.3^37^. The locations of the sampling sites or quadrates were overlaid on the subset of the satellite image that represent the study area using the Leaflet package^38^ in R 4.1.1 platform^39^.

The climate of the site is considered hyper-arid. The highest air temperature (38.1 °C) was recorded during the summer season, while the lowest air temperature (8.2 °C) was recorded during the winter season. The summers are dry, but the average annual mean rainfall in the winter is 0.2 mm/day. The highest average humidity in the winters reaches 58.9%, while the lowest is recorded in the summer season (33.8%). Additionally, the highest mean solar radiation was 28.8 MJ/m^2^/day in the summer months, while the lowest mean solar radiation was 10.9 MJ/m^2^/day in the winter months^40^.

### Water sampling

Water samples were collected from Al-Labene Drain every month for a year extending from March 2019 to February 2020. The study period was categorized into four seasons: Spring extends from March to May, a hot summer extends from June to August, autumn extends from September to November, and a cold winter season extends from December to February. The samples were collected according to APHA (Section 9060 A.2)^41^. The water samples for bacteriological examination were collected in one-liter sterile glass sampling bottles in duplicate. For physicochemical and heavy metal analyses, water samples were collected in polyethylene bottles with a wide mouth, which were cleaned with double distilled water. The samples for heavy metals determination were acidified to less than pH (2) using conc. HCl and HNO_3_. The collected water samples were directly transmitted in an icebox to the laboratory within 2–4 h for analysis.

### Plant sampling, analyses, and rhizofilteration potential

Plant samples were chosen and collected monthly from nine quadrats, each possessing an area of 0.25 m^2^. The quadrats were distributed randomly along the drain in a homogeneous monoculture *P.* *stratiotes* stand. We collected plant samples in plastic bags and separated them into shoots and roots (including stolons) in the lab^36^. The sample’s dry weight was estimated after drying them in an oven at 85 °C until constant weight. Three composite samples of leaves and roots were grind into powders, then 1 g was absorbed in a 20-ml tri-acid mixture of HNO_3_:HClO_4_:HF (1:1:2 V:V:V), filtered, and diluted in 25 mL double de-ionized water^42^. The heavy metals—zinc (Zn), cobalt (Co), and lead (Pb)—were quantitatively analyzed in leaves and roots samples by the atomic absorption spectrophotometer (Shimadzu AA-6200). The accuracy and precision of the metals measurement were estimated using certified reference material 1643e, from the National Institute Standards and Technology (NIST).

Biomass was determined monthly as dry weight/m^2^. The average values of biomass and concentration values of roots and leaves heavy metals were used in the following equation to calculate the rhizofilteration potential (RP), which represents the extracted amount of heavy metals from water in mg_heavy metal_ m^−2^ year^−1^^43^:

1 $${\text{RP = }}\left( {\frac{{{\text{C}}_{{{\text{leaves}}}} {\text{*M}}_{{{\text{leaves}}}} }}{{{\text{M}}_{{{\text{total}}}} }} + \frac{{{\text{C}}_{{{\text{roots}}}} {\text{*M}}_{{{\text{roots}}}} }}{{{\text{M}}_{{{\text{total}}}} }}} \right){\text{*M}}_{{{\text{plant}}}}$$

where M_leaves_ is the dry biomass of leaves (g); M_total_ is the total biomass of leaves and roots (g); C_leaves_ is the heavy metal concentration in leaves (mg kg^−1^); M_roots_ is the roots biomass (g); C_roots_ is the heavy metal concentration in roots (mg kg^−1^); and M_plant_ is the annual mean of *Pistia* yield (g DW/m^2^/year).

### Water physicochemical and heavy metals determination

Water transparency was measured in the field using Secchi disc. Water temperature, dissolved oxygen (DO), electric conductivity (EC) and water pH were measured according to APHA^38^. DO was measured using JENWAY instrument (9500, UK), while, EC was measured using conductivity meter (JENWAY, 4520). Whereas, pH was measured using pH meter (JENWAY, 3510).

Nitrogen (N) was estimated by using the micro-Kjeldahl method, and phosphorous (P) was established using a spectrophotometer (CECIL CE 1021) by applying indophenol blue and molybdenum blue methods, respectively. The samples were deep frozen until further analysis of inorganic elements. Potassium (K) was determined using a flame photometer (FP910-PG Instruments), following Allen^39^. Some heavy metals; zinc (Zn), cobalt (Co), and lead (Pb) were selected according to previously studies, which revealed that *P.* *stratiotes* could be assumed as a biomonitor and bioaccumulator for these particular heavy metals^36,44–47^. Zn, Co, and Pb were determined using an atomic absorption spectrophotometer (Shimadzu AA-6200) following the Standard Methods for Examination of Water and Wastewater^41^. The detection limits for the targeted heavy metals ranged from 0.01 to 0.2 µg/L.

### Quality control

A quality control program was conducted for each experiment series. Blank and analytical standards were used with coefficient for calibration curves higher than 0.99. The Calibration program was verified on each working day by the measurement of one or more standards. Moreover, a random sample was run in triplicate. Laboratory control sample was analyzed with each series of samples. The blanks and standards as analytical grade were obtained from Merck (Germany).

### Microbiological characteristics

Total bacterial counts (TBC) were determined by using the pour plate method on Plate Count Agar, according to APHA/AWWA/WEF^41^ Section (9215B). Briefly, one milliliter of the water sample or its dilution was poured into a sterile Petri dish. Two plates were incubated at 37 ± 0.5 °C for 24 h, and one uninoculated plate was used as a control. Moreover, two plates were incubated at 22 ± 0.5 °C for 48 h, and one uninoculated plate was used as a control.

Total coliform (TC), fecal coliform (FC), and fecal streptococci (FS), as well as *Salmonella* spp., were counted using the membrane filtration (MF) method, following APHA/AWWA/WEF^41^. All bacteriological cultural media were purchased from Difco, USA. TC was determined on an m-Endo agar. Inoculated plates were incubated at 37 ± 0.5 °C for 24 h. The typical red colony, with a metallic sheen, was inoculated on single strength lauryl tryptose broth and brilliant green lactose broth (Difco, USA). The tubes were incubated at 37 ± 0.5 °C for 48 h. The production of acid and gas was recorded as a positive confirmative test for TC.

For FC determination, the membrane filter was transferred on an m-FC agar, and the plates were placed inside waterproof plastic bags and incubated in a water bath at 44.5 ± 0.2 °C for 24 h. For verification, the typical blue colony was inoculated into EC broth tubes. The tubes were incubated at 44.5 ± 0.2 °C for 24 ± 2 h. The production of acid and gas was recorded as a positive confirmative test for FC.

For FS determination, the membrane filter was transferred on m-enterococcus agar plates. The plates were incubated at 37 ± 0.5 °C for 48 ± 3 h. The typical light and dark red colonies were transferred to a brain–heart infusion (BHI) broth and incubated at 37 ± 0.5 °C for 24 ± 2 h. A portion of growth from each BHI broth tube was then streaked onto the surface of a Pfizer selective enterococci (PSE) agar plate. The inverted plates were incubated at 37 ± 0.5 °C for 24 ± 2 h. The development of brownish-black colonies with brown halos formed on the surface of the agar plate confirmed the presence of FS.

*Salmonella* was determined by transferring the membrane filter to the bismuth sulfite agar. The plates were incubated at 37 ± 0.5 °C for 48 h. The typical colonies were black, with or without a metallic sheen. The presumptive colony was streaked onto phenylalanine agar slants, urea broth, and triple sugar iron (TSI) agar for verification. The slants were incubated at 37 ± 0.5 °C for 24 ± 2 h, and a few drops of FeCl_3_ solution (0.5 M) were added. The negative results for both phenylalanine agar and urea test and TSI agar were subjected to serological test using *Salmonella* O Antiserum Poly A-I & VI (Difco, 222641).

### Statistical analysis

In order to assess the significance in seasonal variations in physicochemical, heavy metals, and microbiological parameters of the drainage water, One-way ANOVA has been used, followed by Duncan Test) to determine which seasons differ from the others. ANOVA was performed using SPSS 21.0 software^48^. Forward selection-constrained canonical correspondence analysis (CCA) was performed to determine physicochemical attributes that have the most significant influence on water quality indicators (bacterial abundance and rhizofiltration potential of *P.* *stratiotes*) along the environmental water gradient. CCA was performed using CANOCO 5.0^49^. Pearson correlation coefficients between water variables physicochemical properties, bacterial abundance, *Salmonella* spp., and plant rhizofilteration potential of heavy metals were also calculated using the “corrplot” package in R 3.6.3^50^.

## Results

### Physicochemical and heavy metals in drainage water

There were insignificant seasonal variations in physicochemical analysis of the drainage water, with the exception of dissolved oxygen (DO) which showed significant variation between two groups of seasons: winter and spring representing a group and summer and autumn representing another group (Table 1). Dissolved oxygen was significantly greater (12.36 mg/L) in winter than in autumn (2.74 mg/L). Water transparency varied between 22.5 cm in summer and 18.2 cm in autumn. Water temperature fluctuated during the four seasons between 34.8 °C during summer and 19.8 °C during winter. Water pH varied between 7.9 in spring and 7.1 in autumn. The highest water salinity (EC, 38.9 mS/cm) was recorded during the autumn, while the lowest (16 mS/cm) was in the summer. Regarding inorganic nutrient elements, the highest N value was observed in the winter (23 mg/L), followed by that in the autumn (20.8 mg/L), while the lowest value was observed in summer (11.5 mg/L). There was no obvious seasonal variation in the P; the average values were 10.7, 11.3, 11.9, and 11.9 mg/L during the spring, summer, autumn, and winter seasons, respectively. The F-values of some physicochemical parameters were moderately high. The F-value of DO was the highest and significant. It means that the seasonal variation was relatively high (Table 1).

**Table 1:** Seasonal variation of some physicochemical characteristics and nutrient contents (average ± standard deviation (SD) in water samples collected from drainage water.

Parameters	Unit	Seasons (Average ± SD)				Annual mean	F-value
		Spring	Summer	Autumn	Winter		
Transparency	cm	19.6 ± 0.96	22.5 ± 1.02	18.2 ± 0.97	21.3 ± 2.2	20.4 ± 0.63	2.16
Water temperature	°C	26.76 ± 4.2	32.67 ± 0.34	26.9 ± 2.8	23.88 ± 0.32	27 ± 6.12	2.42
pH	-	7.9 ± 0.27	7.3 ± 0.43	7.1 ± 0.05	7.4 ± 0.22	7.4 ± 0.30	3.53
Dissolved oxygen (DO)	mg/L	11.05 ± 3.7 ^a^	5.16 ± 3.8 ^b^	2.74 ± 0.54 ^b^	12.36 ± 2.25 ^a^	7.83 ± 4.85	7.57*
Electrical conductivity (EC)	mS/cm	22 ± 20	16 ± 16.2	38.9 ± 26.1	22.8 ± 19.7	24.9 ± 4.12	0.22
Nitrogen (N)	mg/L	17.6 ± 2.9	11.5 ± 4.0	20.8 ± 2.3	23 ± 16.1	18 ± 8.5	1.04
Phosphorous (P)	mg/L	10.7 ± 2.54	11.3 ± 0.31	11.90 ± 0.48	11.90 ± 2.93	11.5 ± 1.74	0.24
Lead (Pb)*	mg/L	0.31 ± 0.01	0.30 ± 0.02	0.29 ± 0.008	0.31 ± 0.009	0.30 ± 0.014	1.32
Zinc (Zn)*	mg/L	0.02 ± 0.008	0.02 ± 0.005	0.03 ± 0.005	0.03 ± 0.01	0.03 ± 0.01	0.84
Cobalt (Co)*	mg/L	0.017 ± 0.019	0.022 ± 0.015	0.020 ± 0.016	0.019 ± 0.005	0.019 ± 0.013	0.05

F-values represent repeated measures ANOVA.

*Heavy metals limits in drainage water, according to the Egyptian Law 48/1982, are Pb ≤ 0.5, Zn ≤ 5.0, Co ≤ 2.0 mg/L. *: *p* ≤ 0.05. Small letters “a” and “b” indicate to significant variation of post-hoc Duncan test.

There was a slight seasonal variation in heavy metal values. Pb was insignificantly greater in spring and winter (0.31 mg/L) than in autumn (0.29 mg/L) and summer (0.30 mg/L). Similarly, Zn was insignificantly greater in winter (0.030 mg/L) and autumn (0.028 mg/L) than in summer (0.022 mg/L) and spring (0.020 mg/L). Cobalt was detected at low levels compared with lead and zinc. Cobalt values were 0.017, 0.022, 0.020, and 0.019 mg/L during spring, summer, autumn, and winter seasons, respectively.

### Bacterial counts in drainage water

Total bacterial counts at both 37 °C and 22 °C, total coliform (TC), fecal coliform (FC), fecal streptococci (FS), and *Salmonella* spp. were determined throughout the different seasons (Table 2). The total bacterial counts were slightly higher at 22 °C than 37 °C. The highest values of the total bacterial counts, at 37 °C and 22 °C, were 3.1 × 10^9^ and 3.4 × 10^9^ CFU/mL, respectively, in the summer season. Furthermore, the lowest values of the total bacterial counts, at 37 °C (2.6 × 10^6^ CFU/mL) and 22 °C (3.0 × 10^6^ CFU/mL), were observed in the winter season (Table 2). For example, the total bacterial count attained the highest values in summer (3190 and 3407 CFU/mL at 37 and 22 °C, respectively) but attained the lowest values in winter (2.62 × 10^6^ and 2.98 × 10^6^ CFU/mL at 37 and 22°, respectively). Similarly, the highest counts of TC, FC, and FS were observed during the summer (168.3 × 10^6^, 161.3 × 10^6^ and 139 × 10^6^ CFU/mL, respectively), and the lowest counts were in winter (1.0 × 10^5^, 9.0 × 10^4^, 1.2 × 10^5^ CFU/mL, respectively). Furthermore, *Salmonella* spp. attained significantly greater counts in summer (8.8 × 10^5^ CFU/100 mL) than in both spring and winter (8.0 × 10^3^ CFU/100 mL) (Table 2).

**Table 2:** Seasonal variations of bacterial indicators and *Salmonella* spp. in drainage water.

Season	Bacterial count (× 10^6^, CFU/mL)					
	Total bacterial counts at 37 °C	Total bacterial counts at 22 °C	Total coliform	Fecal coliform	Fecal Streptococci	*Salmonella* spp.
Spring	37.36 ± 37.50	40.33 ± 38.00	2.58 ± 1.82	1.67 ± 1.88	2.36 ± 2.75	0.08 ± 0.06^a^
Summer	3190 ± 503.43	3406.66 ± 527.88	168.33 ± 22.03	161.30 ± 21.71	139 ± 19.22	0.88 ± 0.09^c^
Autumn	395 ± 28.68	453 ± 33.22	25.06 ± 17.97	23.30 ± 16.21	18.10 ± 17.65	0.25 ± 0.29^b^
Winter	2.62 ± 3.09	2.98 ± 3.64	0.10 ± 0.11	0.09 ± 0.10	0.12 ± 0.15	0.08 ± 0.01^a^
F-value	1.1	1.14	1.58	1.5	1.42	18.75**

F-values represent ANOVA and letters (post-hoc Duncan test) for significant levels of seasonal variations.

**p ≤ 0.01. Small letters “a”, “b” and “c” indicate to significant variation of post-hoc Duncan test.

### Rhizofilteration potentials of heavy metals in drainage water

The rhizofiltration potentials (RPs) for the three heavy metals (Pb, Zn, and Co) removed from the drainage water by *P.* *stratiotes* tissues were higher in summer for Pb and Co (219.4 and 17.2 g/m^2^/year, respectively) and autumn for Zn (102.7 g/m^2^/year). The lowest RP value for Pb was observed in the spring (161.6 g/m^2^/year), in the winter and summer (66.6 and 67.4 g/m^2^/year, respectively) for Zn, and in autumn (4.5 g/m^2^/year) for Co (Table 3).

**Table 3:** Seasonal variations of *P.* *stratiotes* rhizofilteration potentials of heavy metals in drainage water.

Season	Rhizofilteration Potential (g/m^2^/year)		
	Pb	Zn	Co
Spring	161.56 ± 53.08	82.38 ± 1.36	14.50 ± 4.85^b^
Summer	219.42 ± 10.67	67.38 ± 11.38	17.21 ± 4.17^c^
Autumn	197.85 ± 5.95	102.72 ± 36.72	4.50 ± 0.542^a^
Winter	188.00 ± 57.16	66.57 ± 8.58	15.87 ± 7.70^b^
F-value	1.1	2.21	3.98*

F-values represent ANOVA and letters (post-hoc Duncan test) for significant levels of seasonal variations.

*p ≤ 0.05. Small letters “a”, “b” and “c” indicate to significant variation of Post-hoc Duncan test.

### Canonical correspondence analysis (CCA)

**Figure 2:**
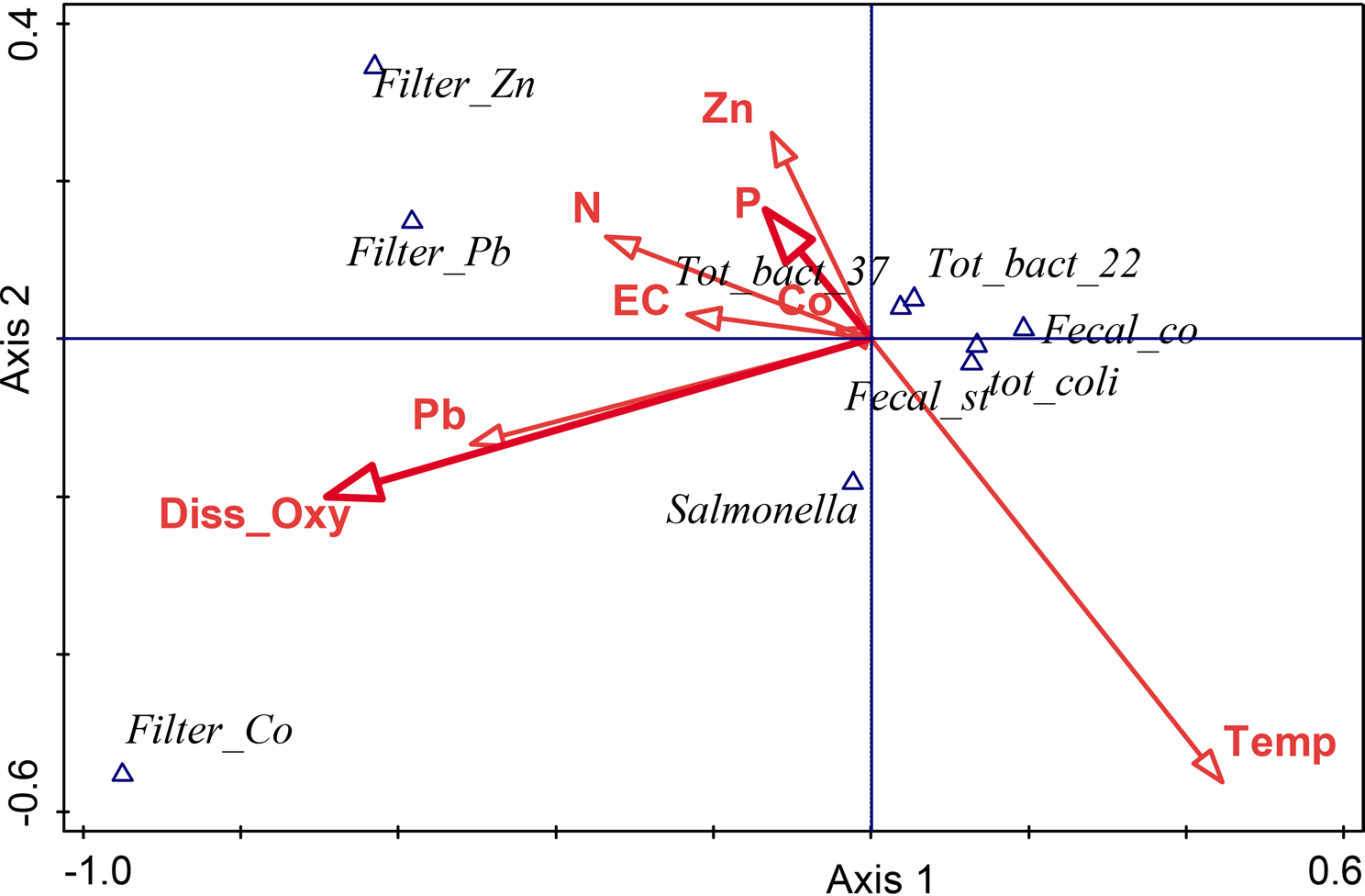
Forward selection results of constrained CCA ordination, with total explained variation by 78%. CCA of bacterial indicators (TBC, at 37 °C and 22 °C, for total coliform, fecal coliform, and fecal *streptococci*), *Salmonella* spp., *P.* *stratiotes* rhizofiltration potential (represented by triangles), and physicochemical water properties (represented by red arrows).

Multivariate analysis forward selection constrained canonical correspondence analysis (CCA) was performed to determine physicochemical attributes that have the most significant influence on water quality indicators (Fig. 2 and Table 4). The results indicated that the second axis of the CCA had a higher explanation for the variation (71.90%) than the first axis (56.61%). The total explained variation was 78%, out of which DO explain 28.5% (36.5%), P explained 12.6% (16.1%), and temperature explained 10.5 (13.5%), but the other factors explained the rest of the variation (26.4%).

**Table 4:** Forward selection results of the constrained CCA and summary of the explained variation.

Water variable	Explains %	Contribution %	Pseudo-F	*p*
**DO**	**28.5**	**36.5**	**4**	**0.032**
N	10.8	13.9	1.6	0.214
Pb	8.5	10.9	1.3	0.298
**P**	**12.6**	**16.1**	**2.2**	**0.096**
Temp	10.5	13.5	2.2	0.106
Co	2.7	3.4	0.5	0.696
EC	1.6	2.1	0.3	0.816
Zn	2.8	3.6	0.4	0.766
**Summary of explained variation**				
Total explained variation	78.00%			
Axis 1 explained variation	56.61			
Axis 2 explained variation	71.9			

The most important explanatory variables, explain % and contribution % are highlighted in bold.

On the other hand, along the second axis of CCA, the abundances of total coliform, fecal streptococci, and pathogenic *Salmonella* spp. were negatively associated with water DO. Additionally, total bacterial counts at 37 °C and 22 °C and fecal coliform abundance along the second axis of CCA were positively associated and affected by water nutrient availability, particularly phosphorus (P) (Fig. 2). The rhizofilteration potential of *P.* *stratiotes* for Zn, as a heavy metal along the second axis, was positively affected by the availability of Zn in the drainage water (Fig. 2).

**Figure 3:**
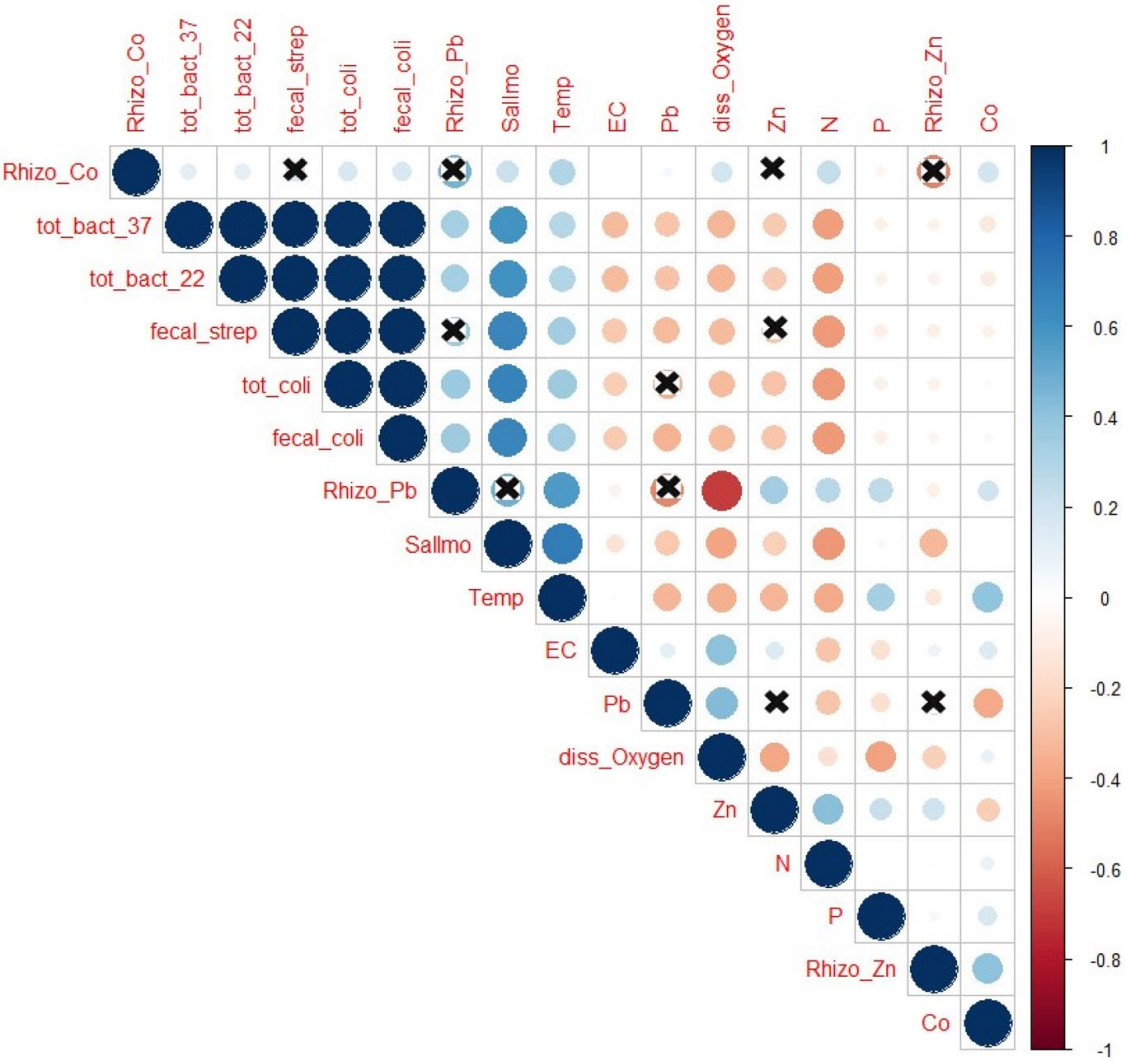
Pearson correlation coefficients (bivariate) between bacterial indicators (TBC at 37 °C and 22 °C for total coliform, fecal coliform, and fecal streptococci), *Salmonella* spp, *P.* *stratiotes* rhizofilteration potential, heavy metals (Pb, Zn, and Co), and physicochemical water properties. The mark “ × ” indicates non-significant correlation (*p* > 0.05).

Figure 3 represents Pearson correlation coefficients (bivariate analysis) that show relationships between physicochemical parameters (temp, EC, DO, N, and P), bacterial counts (TBC, TC, FC, FS, and *Salmonella* spp.), heavy metals in water (Pb, Zn, and Co), and rizofilteration potential of these metals. The results indicated that TBC, at 37 °C and 22 °C, had positive significant correlations with TC, FC, FS, and *Salmonella* spp. Moreover, there were positive significant correlations between temperature and TBC at both 37 °C and 22 °C, TC, FC, FS, and *Salmonella* spp. and rizofilteration for Zn. Furthermore, there were positive correlations between bacterial parameters and rizofilteration potential for Pb. In addition, the rhizofilteration potential of *P.* *stratiotes* for Pb and Co showed positive correlations with water N. The rhizofilteration potential for Zn heavy metal and Zn in the water had a positive correlation (Fig. 3).

Figure 3 represents Pearson correlation coefficients (bivariate analysis) that show relationships between physicochemical parameters (temp, EC, DO, N, and P), bacterial counts (TBC, TC, FC, FS, and *Salmonella* spp.), heavy metals in water (Pb, Zn, and Co), and rizofilteration potential of these metals. The results indicated that TBC, at 37 °C and 22 °C, had positive significant correlations with TC, FC, FS, and *Salmonella* spp. Moreover, there were positive significant correlations between temperature and TBC at both 37 °C and 22 °C, TC, FC, FS, and *Salmonella* spp. and rizofilteration for Zn. Furthermore, there were positive correlations between bacterial parameters and rizofilteration potential for Pb. In addition, the rhizofilteration potential of *P.* *stratiotes* for Pb and Co showed positive correlations with water N. The rhizofilteration potential for Zn heavy metal and Zn in the water had a positive correlation (Fig. 3).

## Discussion

Drains in Giza Governorate receive a high quantity of agricultural wastewater, in addition to the untreated and insufficiently treated municipal wastewater from Abou-Rawash primary wastewater treatment plant and Zennin secondary wastewater treatment plant. The two wastewater treatment plants serve more than 6 million people. All of the treated wastewater is finally discharged into the River Nile (Rosetta Branch) through numerous drains^51^. Therefore, this study focused on seasonal variation of chemical and microbial pollutants and rhizofiltration potentials of *P.* *stratiotes* in the drainage water.

The physicochemical features of the water ecosystem have an important role in controlling water quality and can alter the diversity and community of water biota^52^. Each water body has specific physicochemical parameters, which are generally determined by climatic, geomorphologic, and geochemical situations prevailing in the drainage basin and the underlying aquifer^53^. In this study, there was low average water transparency (20.4 cm). However, there were insignificant differences in water transparency between seasons. The higher water transparency in summer (22.5 cm) than in spring (19.6 cm), and autumn (18.2 cm) could be attributed to an increase in water flow and anthropogenic activity in the summer season. Water transparency is considered a beneficial indicator of water quality and also is commonly used to detect long-term variations in water quality and eutrophication of water ecosystems^54^. In the present study, water temperature was also higher in summer and spring than in winter and autumn. Lower water transparency, together with higher temperatures during summer, could encourage eutrophication. Such eutrophication could be a main reason for an increase in pH in summer than in other seasons. It has been reported that the variation in water pH and temperature may be attributed to photosynthetic activities and respiration of aquatic plants and animals^53^.

It is well known that the increase in air movement increases DO^55^. Moreover, low temperature in the winter season possibly raises the ability of water to hold more DO^56^. Rounds et al. (2013)^57^ also found that temperature has a direct effect on the amount of the DO. In addition, higher eutrophication during summer increase the amounts of organic matter in the warm season, resulting in an increase in indigenous microorganisms that consume DO during organic matter biodegradation^55^. Similar results were reported in other drainage water plants (El-Rahawy Drain) in the Giza area that had low DO, high turbidity, and high organic and inorganic pollutants^19^. The lower concentration of DO in summer (5.16 mg/L) and autumn (2.74 mg/L) could affect the activity of biota in the study drain. It was reported that a DO content of less than 5 mg/L might negatively affect the survival of biological communities. In addition, DO concentrations of less than 2 mg/L may well result in the death of most fish species^58^. The average value of DO in the El-Rahawy Drain, which also receives wastewater, was 4.5 mg/L^59^.

Measurements of EC remain a suitable measure for temporal variations in surface water dissolved solids (TDS) and the majority of ions^58^. The electrical conductivity (EC) of most freshwater bodies ranged from approximately 0.1 to 1.0 mS/cm; occasionally, it may exceed 1.0 mS/cm, particularly in polluted water. The year average of EC in the study drain was 24.9 mS/cm, with some seasonal variation ranging between 38.9 mS/cm in autumn and 16 mS/cm in summer. However, Galal et al.^36^ found that the highest EC value in the Al-Sero Drain in Giza, Egypt, was in January (winter, 42.8 mS/cm), while the lowest was in November (autumn, 3.3 mS/cm). They mentioned the low EC in autumn relative to the higher water level, which reached up to 180.9 cm^40^. The higher EC in the study drain could be attributed to high concentrations of soluble salts, cations, and anions of the agricultural, sewage, and industrial discharged wastewater^60^.

Macronutrients, such as N and P, are imperative components for plant growth and advancement and positively contribute to crop development. Unwarranted supplements in the water system can result in a wellspring of groundwater contamination, similar to eutrophication in surface water^61^. N is one of the main pollutants in wastewater; it can affect DO levels of receiving water and cause toxicity to aquatic organisms. Nitrogen exists in wastewater in both organic (e.g., peptide, protein, urea, and uric acids) and inorganic forms (e.g., ammonia, nitrate, and nitrite)^62^. In this study, the annual average N content was 18 mg/L; the highest was in the winter, while the lowest was in the summer. This may be attributed to the fact that the high temperature of summer enhances denitrification processes that reduce water N. Consequently, N removal in the summer is higher than in winter^63^. Phosphorus frequently occurs in the form of phosphate (PO_4_^3−^) in surface water. Potential sources of phosphate contamination include fertilizers, soil erosion, domestic and industrial wastewaters, and animal waste^64^. The results of this study showed that the annual average P was considerable (11.5 mg/L), with little variation between the seasons. This indicates that the presence of a continuous pollution source for P all over the year. The lowest P was observed during spring (10.7 mg/L), while the highest p values were observed during autumn and winter (11.9 mg/L). Otherwise, Doja^65^ observed that the removal of P decreases because algae grow slower in the winter season.

Heavy metals are considered among the main pollutants that harm humans and animals. They are nonbiodegradable elements and can accumulate in living organisms^66^. Industrial wastewater contains, among others, lead, zinc, copper, cobalt, and chromium^67^. Detectable heavy metals (Pb, Zn, and Co) concentrations in Al-Labene Drain did not exceed the permissible limits of Egyptian law (Act 8 of 1982). In addition, there were no noticeable seasonal variations in heavy metal levels over the year. This indicates that the industrial activities that discharge their water in Al-Labene Drain have lower levels of the three studied heavy metals. In addition, the higher water flow in Al-Labene Drain^59^ dilutes most pollutants, including heavy metals. The heavy metals are not biodegradable, and their presence in the food chain through several pathways may be accumulated in different organs of human beings or animals^68^. Therefore, it is important to monitor heavy metals in water bodies, even in low concentrations.

Waterborne diseases have been a major global health problem. Pathogens are constantly released to water drains at different densities from infected humans, pets, and animals. Municipal wastewater and storm-water runoff developments are the conduits for the passage of pathogens into surface water^69,70^. Surface waters, such as rivers, drains, and lakes, act as natural reservoirs of various microbial populations and also play an essential role in the transmission of enteric pathogens such as pathogenic *E.* *coli*, *Salmonella* spp., *Vibrio* *cholerae*, rotaviruses, and helminths^17,71,72^. In the present study, bacterial indicators (TBC, TC, FC, and FS) and *Salmonella* spp. were recorded with considerable counts in the water of the Al-Labene Drain over the year. The highest bacterial indicators and *Salmonella* spp. counts were observed during the summer. One possible explanation could be the increase in anthropogenic activities, especially during the summer. In addition, the municipal wastewater flowing to the Al-Labene Drain from Abou-Rawash WWTP possibly has high levels of organic pollutants. It has been reported that Al-Labene Drain receives more than 200,000 m^3^ of the raw wastewater daily from Abou-Rawash wastewater treatment without even undergoing primary treatment^73^. In addition, to the role of the raise in temperature during summer in increasing the bacterial counts^74^. Over the year, fecal coliform counts exceeded the permissible limits prescribed by Egyptian law (Act 48 of 1982), indicating that Al-Labene Drain water is not suitable to be reused for irrigation purposes. The use of Al-Labene Drain water without further treatment could directly impact human and animals’ health.

Aquatic macrophytes tolerate life in polluted water and accumulate heavy metals from the surrounding environment^75^. In the present study, *P.* *stratiotes* accumulated considerable amounts of Pb, Zn, and Co (188, 66.6, and 15.9 g/m^2^/year). The highest rhizofiltration potential was in the summer (for Pb and Co) and autumn (for Zn). It has been reported that *P.* *stratiotes* can enhance nutrient removal through the interception of inorganic and organic particulates and the creation of an oxidized rhizosphere^36^. In addition, Nanda and Abraham^76^ found that *P.* *stratiotes* can accumulate significant levels of heavy metals, with more than 1000 mg/kg in its roots that were significantly higher than in leaves. This finding was also confirmed by Mufarrege et al.^77^ and Galal et al.^36^. Interestingly, RP amounts were higher for Pb and Zn than for Co, which exceeded regulatory safety levels for concentrations in other plants^42,78^.

## Conclusions

Al-Labene Drain receives huge amounts of agricultural, industrial, partially treated, and untreated domestic wastewater, which negatively impact water quality and in turn, threatens human and animal health. The findings showed seasonal variation in the physicochemical and bacteriological parameters of drain water. The highest total bacterial counts, at 37 °C and 22 °C, for total coliform, fecal coliform, fecal *Streptococci*, and *Salmonella* spp. in the drainage water were observed during the summer season. The high rhizofiltration potential of *P.* *stratiotes* for heavy metals (Pb, Zn and Co) indicates its potential in phytoremediation of polluted sites. High levels of heavy metals can harm *P.* *stratiotes*; therefore, it is recommended to frequently collect, burn, and dump ash in safe landfills. Based on the outcomes obtained, more precautions need to be considered regarding the release of surplus untreated wastewater from the Abou-Rawash WWTP, which is considered the main pollution source for several drains, including the Al-Labene Drain. Treatment of wastewater by phytoremediation can contribute to achieving sustainability in water resources.

## Data Availability

The data presented in this study are available on request from the corresponding author.
